# Weight Reduction Through a Digital Nutrition and Food Purchasing Platform Among Users With Obesity: Longitudinal Study

**DOI:** 10.2196/19634

**Published:** 2020-09-02

**Authors:** Emily A Hu, Viet Nguyen, Jason Langheier, Dexter Shurney

**Affiliations:** 1 Zipongo, Inc, DBA Foodsmart San Francisco, CA United States; 2 Department of Epidemiology Johns Hopkins Bloomberg School of Public Health Baltimore, MD United States; 3 American College of Lifestyle Medicine Chesterfield, MO United States

**Keywords:** digital, nutrition, meal planning, weight loss, obese, food environment, food ordering, food purchasing, behavioral economics, behavior change, eating behavior, mHealth, app

## Abstract

**Background:**

Digital nutrition apps that monitor or provide recommendations on diet have been found to be effective in behavior change and weight reduction among individuals with obesity. However, there is less evidence on how integration of personalized nutrition recommendations and changing the food purchasing environment through online meal planning and grocery delivery, meal kits, and grocery incentives impacts weight loss among individuals with obesity.

**Objective:**

The objective of this observational longitudinal study was to examine weight loss and predictors of weight loss among individuals with obesity who are users of a digital nutrition platform that integrates tools to provide nutrition recommendations and changes in the food purchasing environment grounded in behavioral theory.

**Methods:**

We included 8977 adults with obesity who used the digital Foodsmart platform, created by Zipongo, Inc, DBA Foodsmart between January 2013 and April 2020. We retrospectively analyzed user characteristics and their associations with weight loss. Participants reported age, gender, height, at least 2 measures of weight, and usual dietary intake. Healthy Diet Score, a score to measure overall diet quality, was calculated based on responses to a food frequency questionnaire. We used paired *t* tests to compare differences in baseline and final weights and baseline and final Healthy Diet Scores. We used univariate and multivariate logistic regression models to estimate odds ratios and 95% CI of achieving 5% weight loss by gender, age, baseline BMI, Healthy Diet Score, change in Healthy Diet Score, and duration of enrollment. We conducted stratified analyses to examine mean percent weight change by enrollment duration and gender, age, baseline BMI, and change in Healthy Diet Score.

**Results:**

Over a median (IQR) of 9.9 (0.03-54.7) months of enrollment, 59% of participants lost weight. Of the participants who used the Foodsmart platform for at least 24 months, 33.3% achieved 5% weight loss. In the fully adjusted logistic regression model, we found that baseline BMI (OR 1.02, 95% CI 1.02-1.03; *P*<.001), baseline Healthy Diet Score (OR 1.06, 95% CI 1.05-1.08; *P*<.001), greater change in Healthy Diet Score (OR 1.12, 95% CI 1.11-1.14; *P*<.001), and enrollment length (OR 1.28, 95% CI 1.23-1.32; *P*<.001) were all significantly associated with higher odds of achieving at least 5% weight loss.

**Conclusions:**

This study found that a digital app that provides personalized nutrition recommendations and change in one’s food purchasing environment appears to be successful in meaningfully reducing weight among individuals with obesity.

## Introduction

The increasing prevalence of obesity worldwide is a critical public health problem [1,2]. In the United States, about 39.6% of adults 20 and older were considered obese in the years 2015-2016, and the prevalence is projected to increase [3]. Overweight and obesity pose serious health challenges as they are strong risk factors for cardiovascular disease, type 2 diabetes, chronic kidney disease, many cancers, and mortality [1,4,5].

The prevention and management of obesity are extremely necessary given the potential health and cost consequences [6]. For decades, there has been mounting evidence from large trials such as the Diabetes Prevention Program (DPP) showing that change in lifestyle, often related to weight reduction, can have dramatic effects on health and chronic disease [7-12]. However, interventions like DPP have failed to sustain weight loss more than 18-24 months and can be costly due to coaching time and the cycles of losing and regaining weight [13,14].

Digital health technologies that incorporate nutrition education and monitoring have gained increasing popularity to change and manage dietary choices [15-17]. Previous studies on mobile apps to improve nutrition are promising as their results indicate that digital nutrition interventions may be effective in changing dietary behavior to improve weight, glucose, and blood pressure among healthy individuals and people at risk of or with chronic disease [18-21]. While many of these apps provide general diet recommendations, few apps have a decision engine capable of providing personalized dietary advice, meal planning assistance, and online grocery delivery to users [16].

Meal planning and at-home cooking have been found to be associated with greater adherence to dietary guidelines, increased fruit and vegetable intake, and greater variety of foods consumed [22,23]. Meal planning behaviors, including frequency of planning meals ahead of time, grocery shopping and cooking, have been associated with lower likelihood of obesity in men and women [23].

To our knowledge, no studies with meaningful scale have examined the effect of a digital technology that provides personalized healthy meal plans and changes in the food purchasing environment (through online grocery shopping, purchase discounts, delivery, and meal kits) on health outcomes. There is a need for additional evidence on how digital technologies that alter behavioral economics, such as food purchasing, might play a role in improving diet and driving more cost-effective health outcomes for individuals with obesity.

Our goal was to conduct an observational longitudinal study leveraging existing data from a digital nutrition platform to investigate the effectiveness of a personalized nutrition, meal planning, and food purchasing program on weight loss among individuals with obesity.

## Methods

### Study Population

The current study is a longitudinal analysis of 8977 adults with obesity (aged 18 to 80 years, living in the United States) who enrolled in the Foodsmart platform. Of the 888,999 users who had enrolled up to April 2020, we excluded individuals who did not report weight (n=562,276), those who reported extreme values for height (<54 in or >78 in, ie, <1.37 m or >1.98 m) or weight (<60 lb or >400 lb, ie, <27.2 kg or >181.4 kg) (n=25,946), and those who were not obese (BMI<30 kg/m^2^) at the time of enrollment (n=200,308). We further excluded those who reported BMI after more than 3 days from joining Foodsmart, those whose BMI changed more than 15 kg/m^2^ in less than 10 months, and participants with greater than 16% weight change in less than 1 month (n=13,548). We additionally excluded those who did not report weight at least 2 times (n=9023) and participants who did not fill out the Nutriquiz survey twice (n=68,921).

### Foodsmart Platform

**Figure 1:**
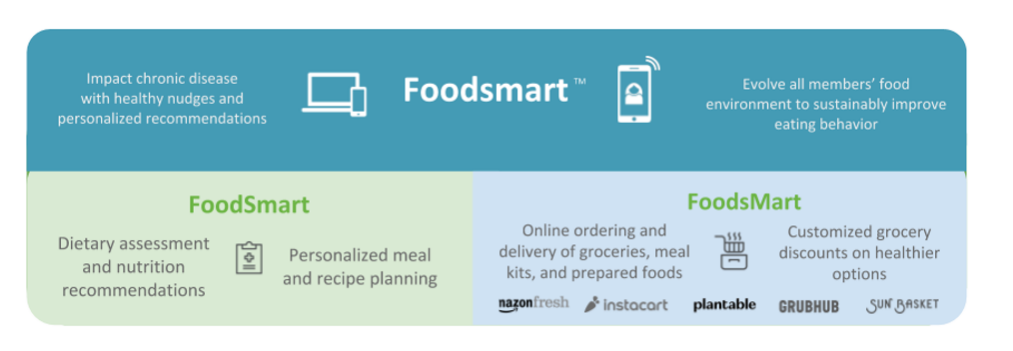
Components and tools of Foodsmart.

Foodsmart (Zipongo Inc DBA Foodsmart) built a digital nutrition platform called Foodsmart that is designed to make healthier dietary choices simple and sustainable through personalization of nutrition and meal/recipe recommendations by creating a food purchasing environment that provides healthy options for all people, whether they enjoy cooking, prefer to use meal kits, or prefer prepared meals. Foodsmart is made up of 2 components (FoodSmart and FoodsMart) with self-directed tools that drive knowledge, motivation, and planning to make it easier and more affordable to prepare tasty, healthy food at home (Figure 1).

The platform was developed using Prochaska’s Theory of Change model as the baseline theory supplemented with elements from the Behaviour Change Techniques Taxonomy [24,25] (Table 1). The tools from both FoodSmart and FoodsMart are designed to target all stages (pre-contemplation, contemplation, preparation, action, and maintenance) of behavior change in healthier eating. These tools encourage users to reflect and assess their dietary habits with Nutriquiz, helping create a specific plan for users to eat healthier daily, offering tools to purchase healthy foods, and providing incentives and communication to maintain healthy behaviors.

**Table 1:** Foodsmart platform components and tools linked with behavior change stages and techniques.

Foodsmart components and tools		Stages of change [24]	Behaviour Change Techniques [25]
**FoodSmart**			
	Nutriquiz dietary assessment and re-assessment and dietary recommendations	Pre-contemplation: Encourages user to think about dietary habits Contemplation: Results encourage users to think of changes to make in diet Preparation: Helps create a specific plan on which foods to change Maintenance: Monitors progress by re-taking Nutriquiz	Provide information about behavioral health link Prompt intention formation Prompt specific goal setting Prompt self-monitoring of behavior Prompt self-monitoring of performance Provide feedback on performance Provide opportunities for social comparison
	Family meal planning (recipe recommendations for each meal through linkage to recipe database)	Preparation: Assists the user in making a plan to cook Action: Automatically loads recipe ingredients to grocery list	Prompt barrier identification Set graded tasks Provide instruction Stress management Time management
	Social liking and commenting of recipes	Preparation: Prepares the user to cook by browsing and interacting with recipes; also builds social support to be successful	Plan social support or social change
	Enrollment and activation marketing (incentives, enrollment emails, newsletters)	Pre-contemplation: Enrollment emails and newsletters create awareness of capabilities Contemplation: Emails encourage people to activate certain features based on needs; incentives provide contingent awards for participating Maintenance: Newsletters and emails to encourage people to keep using platform	Provide general encouragement Provide contingent awards
**FoodsMart (advertising of unhealthy foods is filtered out)**			
	Online grocery list and food ordering (including prepared meals and meal kits)	Preparation: Online grocery list helps identify barriers Action: Online food ordering helps stress and time management Maintenance: Once a user practices and demonstrates the behavior of creating a list online, more likely to maintain online food ordering	Default behavioral economics Prompt barrier identification Prompt practice Set graded tasks Provide instruction Model or demonstrate the behavior Stress management Time management
	Food discounts and incentives	Contemplation: Incentives provide contingent awards for participating Preparation: Discounts allow for budgeting before grocery shopping Action: Makes it feasible to buy healthy food that otherwise can’t afford Maintenance: Discounts and incentives encourage continual usage by helping with stress and time management	Behavioral economics Stress management Time management

The first component is FoodSmart, which contains the in-app Nutriquiz, a dietary assessment (based on the National Cancer Institute’s Diet History Questionnaire). Users can take Nutriquiz to report their dietary habits, which provides immediate and specific feedback on aspects of their diet to improve on as well as personalized meal and recipe planning based on the Nutriquiz results. Over time, users can retake the Nutriquiz assessment to monitor their own progress related to specific nutrients and food groups as well as their progress on health goals, like weight. The second component is FoodsMart, which helps reset one’s default behavioral economics by altering the food purchasing environment. This is achieved through personalized meal plan conversion to a grocery list and integrated online ordering and delivery of groceries, meal kits, and prepared foods, where food advertising paid for by food manufacturers is removed and replaced with nudges to make healthier substitutions that align with user preferences and their personalized meal planning. Customized grocery discounts on healthier options help the user save money and further nudge the user to make healthier choices. The Foodsmart platform has been in use since 2013; and 90% of users enrolled in 2017 or later, after most of the major content and design changes to the platform were made (Multimedia Appendix 1). The platform has evolved over time, with the most significant change being the addition of grocery and food ordering in the last few years. The product is available through certain health plans and employers who have signed up for Foodsmart, and they can provide this product as an option or benefit for their members/employees to enroll in. It is available to be used on the web, iOS, and Android operating systems.

### Measurements

All data were self-reported through the Foodsmart app during the study period. When users created their account, they were prompted to fill out a survey created by Foodsmart called Nutriquiz, a 53-item food frequency questionnaire adapted from the National Cancer Institute Diet History Questionnaire, which has been previously validated [26]. The questionnaire ascertains biological sex, birth date, weight, and usual intake of food groups and nutrients. For example, it asks, “How often do you eat fruit?” Possible responses include “never,” “monthly,” “weekly,” and “daily.” Other food groups assessed included vegetables, whole grains, proteins, carbohydrates, fats, fiber, sodium, and water. Foodsmart’s research team created a healthy diet score called Healthy Diet Score, which is based on the Alternative Healthy Eating Index-2010 (AHEI-2010) and the Commonwealth Scientific and Industrial Research Organization (CSIRO) Healthy Diet Score [27,28]. Similar to the AHEI-2010, the Healthy Diet Score includes fruits, vegetables, and sodium components; and each component is scored 1-10 using absolute cutoffs. In order to keep the score concise, the Healthy Diet Score combined macronutrient components in a similar fashion to the CSIRO Healthy Diet Score, which includes only 1 category each for protein, carbohydrates, and fats. Additionally, it has a component for fluids, which was modified to be hydration in the Healthy Diet Score since percent fluid intake is more relevant than total quantity of fluids. For calculation of the Healthy Diet Score, participants were assigned a score from 0-10 for 7 components: fruit, vegetable, protein ratio (white meat/vegetarian protein to red/processed meat), carbohydrate ratio (total fiber to total carbohydrate), fat ratio (polyunsaturated to saturated/trans fats), sodium, and hydration (percent of daily fluid goal). Higher scores indicated healthier habits. A total Healthy Diet Score to evaluate overall diet quality was calculated by summing the scores of the 7 components, with the total possible score ranging from 0 to 70. Change in Healthy Diet Score was calculated as the difference between the first Healthy Diet Score and the last Healthy Diet Score. We compared participants whose Healthy Diet Score decreased or was stable (no improvement in diet quality) with those participants whose Healthy Diet Score increased (improvement in diet quality) between the first and last report. We collapsed decreased and stable categories due to a low number of participants in the stable category.

Participants were asked to add weight and height data when they joined and could update their weight at any time during usage of the platform. Baseline BMI was calculated as first weight entry in kilograms divided by height in square meters (kg/m^2^). We categorized participants by baseline obesity class. Class 1 obesity was defined as a BMI between 30 to 34.9 kg/m^2^; class 2 was defined as a BMI of 35 to 39.9 kg/m^2^; and class 3 was defined as a BMI of 40 kg/m^2^ or higher. To calculate a change in weight, we subtracted the last reported weight from the first reported weight. Our primary outcome was 5% or greater weight loss, which has been found to be clinically significant and associated with improvements in cardiometabolic risk factors such as lipid profile and insulin sensitivity [9,12,29,30].

Duration of enrollment (in months) in Foodsmart was calculated as follows: the number of days between the date on which participants initially entered their weight and the date on which they entered their last follow-up weight was calculated and divided by a factor of 30.437 to convert to months. We classified participants into enrollment categories of 0 to 6 months, greater than 6 to 12 months, greater than 12 to 18 months, greater than 18 to 24 months, and 24 months or greater. For stratified analyses, we collapsed the greater than 18 to 24 months and 24 months or greater into one category of greater than 18 months.

### Statistical Analysis

Descriptive statistics were used to examine baseline characteristics of the total study population and to compare whether participants lost at least 5% of their initial body weight. Categorical variables were reported as frequencies (%) and continuous variables were reported as mean (SD). Chi-square tests and analysis of variance (ANOVA) tests were used to test differences for categorical and continuous variables, respectively, between participants who achieved 5% weight loss and participants who did not.

We examined the change in weight and Healthy Diet Score by using paired *t* tests between baseline and final weights and Healthy Diet Scores of participants. We then used univariate logistic regression models to estimate odds ratios and 95% CI between achievement of 5% weight loss and independent variables: gender, age, baseline BMI, baseline Healthy Diet Score (per 2-point increase), change in Healthy Diet Score (per 2-point increase), and length of enrollment (per 6 months). Multivariate logistic regression models were adjusted for variables that were statistically significant to investigate independent associations with achievement of 5% weight loss.

Further, we conducted stratified analyses to examine differences in percent weight change by enrollment length and stratified by gender, age category, BMI class, and change in Healthy Diet Score. We used bar graphs to visualize differences and ANOVA tests to statistically test for differences, using a Bonferroni-corrected *P* value of .0031 to account for multiple comparisons.

We considered a *P* value smaller than .05 to be significant for all tests except for the ANOVA tests used for detecting differences in stratified groups. R studio version 1.2.5033 and Stata version 16 (StataCorp) were used for all analyses.

The study was declared exempt from institutional review board oversight by the Pearl Institutional Review Board given the retrospective design of the study and less than minimal risk to participants.

## Results

### Participant Characteristics

Baseline characteristics of the total study sample and stratified by whether participants achieved 5% weight loss are shown in Table 2. Categorical variables were reported as frequencies (%) and continuous variables were reported as mean (SD).

**Table 2:** Baseline characteristics of Foodsmart users.

		Total (N=8977)	Did not lose ≥5% of initial weight (n=6838)	Lost ≥5% of initial weight (n=2139)	*P* value^a^
Male, %		20.1	20.1	20.2	.9
Age, years, mean (SD)		46.6 (11.0)	46.3 (11.0)	47.3 (11.0)	.01
Height, m, mean (SD)		1.7 (0.1)	1.7 (0.1)	1.7 (0.1)	.1
Baseline weight, kg, mean (SD)		101.7 (18.3)	101.2 (18.1)	103.4 (18.9)	<.001
Baseline BMI, kg/m^2^, mean (SD)		36.3 (5.6)	36.2 (5.6)	36.8 (5.8)	<.001
**Obesity category**					**<.001**
	Obesity class 1 (30-34.9 kg/m^2^), %	52.3	53.4	48.7	
	Obesity class 2 (35-39.9 kg/m^2^), %	26.7	26.6	27.0	
	Obesity class 3 (≥40 kg/m^2^), %	21.1	20.1	24.4	
Baseline Healthy Diet Score (0-70), mean (SD)		30.3 (8.6)	30.2 (8.6)	30.6 (8.6)	.1
Final Healthy Diet Score (0-70), mean (SD)		32.6 (8.5)	31.9 (8.5)	34.8 (8.3)	<.001
Change in Healthy Diet Score, mean (SD)		2.3 (7.5)	1.7 (7.3)	4.2 (7.9)	<.001
Enrollment length, months, mean (SD)		11.4 (8.3)	10.7 (8.1)	13.6 (8.3)	<.001
Weight change, %, mean (SD)		–1.5 (7.5)	1.5 (4.9)	–11.1 (6.4)	<.001
Weight change, kg, mean (SD)		–1.7 (7.9)	1.4 (5.0)	–11.6 (7.7)	<.001

^a^Chi-square tests and analysis of variances tests were used to test differences for categorical and continuous variables.

Using *t* tests, we found that final BMI was significantly lower than the baseline BMI (*P*<.001); and final Healthy Diet Score was significantly higher than the baseline Healthy Diet Score (*P*<.001). In total, 59% of participants reported losing weight, and 24% reported loss of at least 5% of their initial weight. Compared to participants who did not lose at least 5% of their initial weight, those who did were more likely to be slightly older, have a slightly higher baseline weight, be classified as obesity class 3, have a higher change in Healthy Diet Score, and be enrolled in the Foodsmart program longer (Table 2). Their baseline Healthy Diet Scores were comparable.

### Predictors of At Least 5% Weight Loss

**Figure 2:**
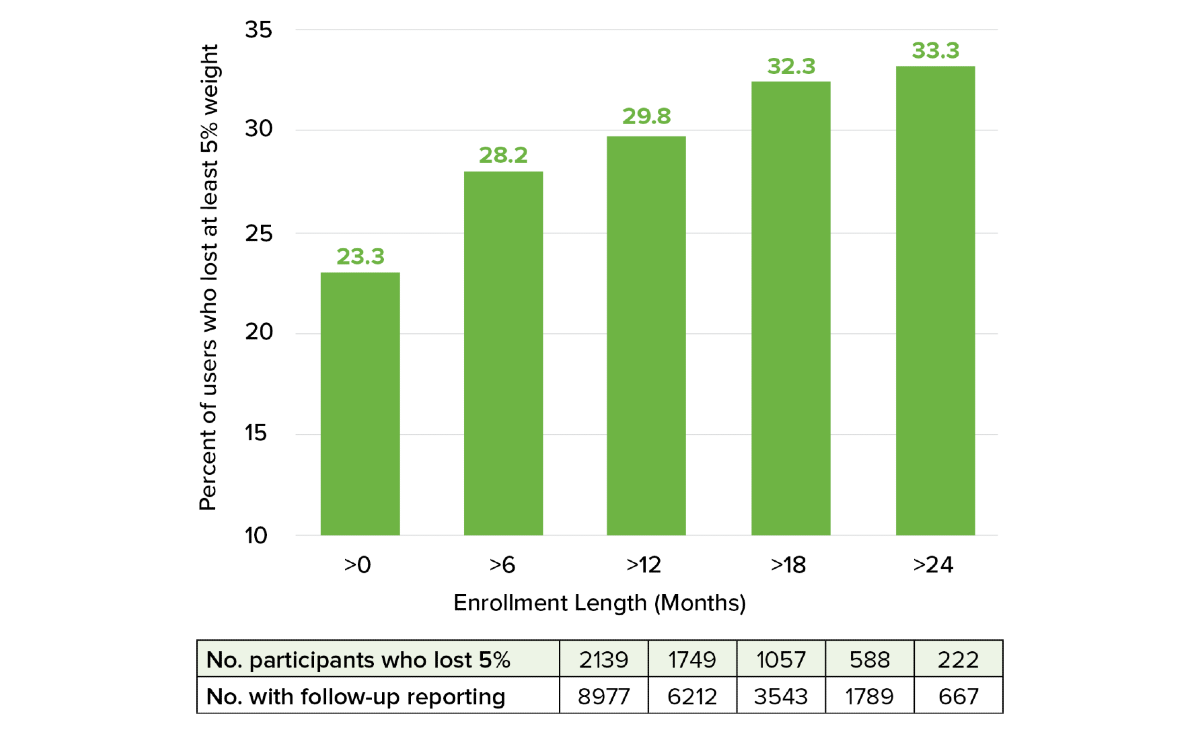
Percent of users who lost at least 5% of initial weight by cumulative enrollment duration.

The percentage of users who lost at least 5% of their initial weight increased with longer enrollment duration (Figure 2).

Individual variables contributing to at least 5% weight loss were assessed using univariate logistic regression models (Table 3).

We collectively analyzed the variables and 5% weight loss in a multivariate logistic regression model. Baseline BMI, baseline Healthy Diet Score, greater change in Healthy Diet Score, and enrollment length were all directly associated with higher odds of achieving at least 5% weight loss.

**Table 3:** Factors contributing to at least 5% weight loss in univariate and multivariate logistic regression models.

	Univariate OR (95% CI)	*P* value	Multivariate OR (95% CI)	*P* value
Gender (male)	1.01 (0.89-1.14)	.9	1.03 (0.91-1.17)	.7
Age, years	1.01 (1.00-1.01)	<.001	1.00 (1.00-1.01)	.4
Baseline BMI, kg/m^2^	1.02 (1.01-1.03)	<.001	1.02 (1.02-1.03)	<.001
Baseline Healthy Diet Score, per 2-point increase	1.01 (1.00-1.02)	<.001	1.06 (1.05-1.08)	<.001
Change in Healthy Diet Score, per 2-point increase	1.09 (1.08-1.11)	<.001	1.12 (1.11-1.14)	<.001
Enrollment length, per 6 months	1.28 (1.24-1.32)	<.001	1.28 (1.23-1.32)	<.001

### Stratified Analyses

**Figure 3:**
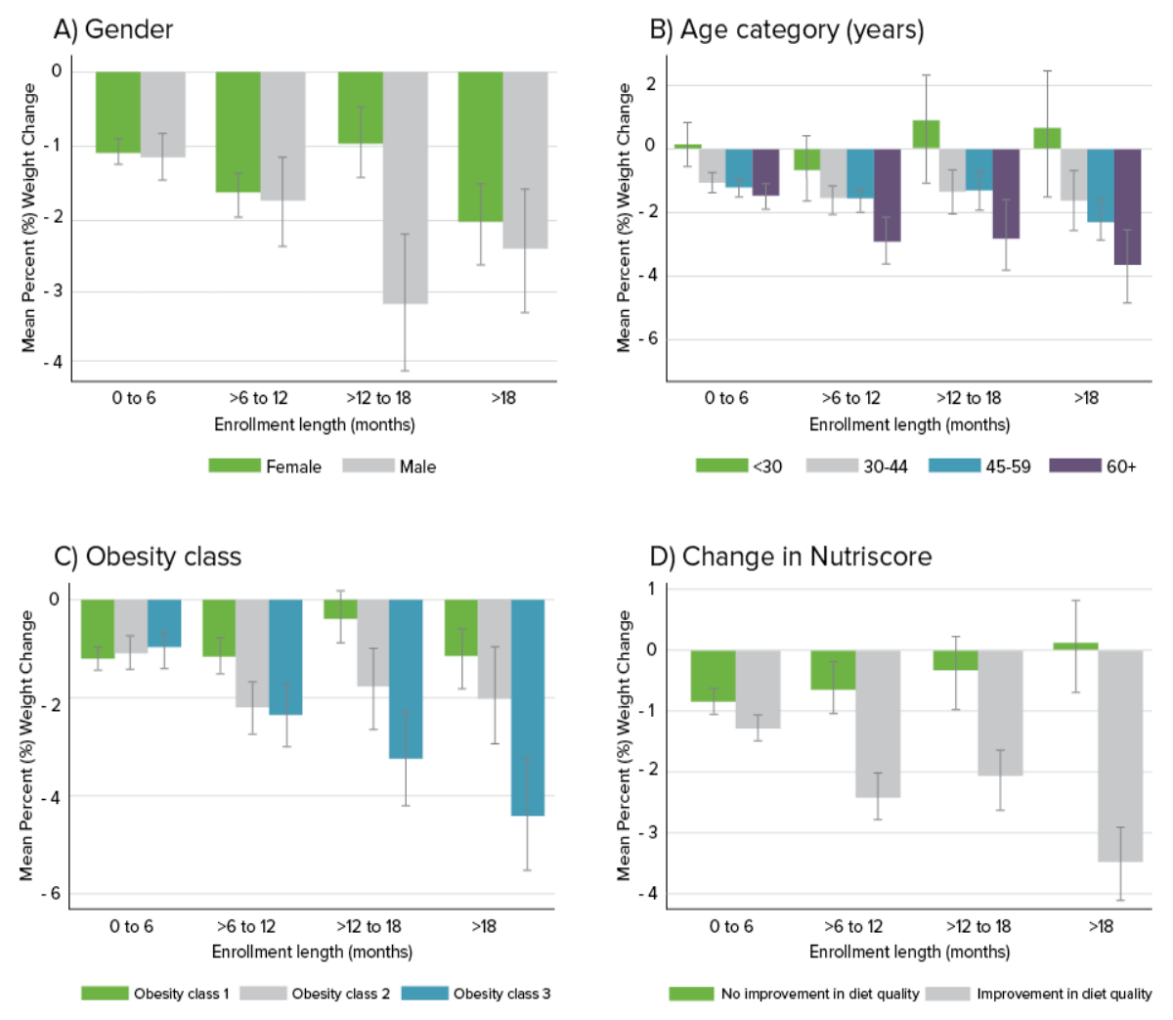
Mean (SD) percent weight change stratified by enrollment length and A) gender, B) age category, C) baseline obesity class, and D) change in Healthy Diet Score. Gray error bars indicate standard deviations of the mean; * indicates a statistically significant difference between groups assessed using ANOVA tests and a Bonferroni-corrected *P* value of .0031 to adjust for multiple comparisons.

Figure 3A-D shows the mean percent weight change stratified by enrollment length category (less than 6 months, greater than 6 months to 12 months, greater than 12 months to 18 months, greater than 18 months) and by gender, age category, BMI category, and change in Healthy Diet Score (increase vs stayed the same or decrease).

While male users experienced, on average, greater weight loss compared to female users, the difference was much more pronounced among participants who were enrolled for 12-18 months. We also observed that when stratified by age, participants who were older experienced greater weight loss compared with those who were younger in a dose-response relationship. Participants in the highest age category of 60 and older lost the most weight, and this association became more robust with longer enrollment duration. Similarly, when stratified by baseline obesity class, participants who were in obesity class 3 had the largest improvements in weight, followed by obesity class 2 and then obesity class 1. The associations strengthened with enrollment duration. Participants who increased their Healthy Diet Score between their first and last reports of dietary intake experienced greater weight loss compared with participants whose Healthy Diet Score stayed the same or decreased.

## Discussion

In the present study of 8977 Foodsmart platform users with obesity, we found that 59% of participants reported a decrease in weight, and 24% reported at least 5% weight loss of their initial weight while enrolled in the program; median (IQR) duration of follow-up was 9.9 months (0.03-54.7). Baseline BMI, baseline Healthy Diet Score, change in Healthy Diet Score, and longer duration of enrollment were all associated with higher odds of achieving 5% or greater weight loss. Age and gender were not associated with at least 5% weight loss. We found that the percentage of participants achieving 5% weight loss increased with enrollment duration. These findings suggest that Foodsmart platform users with obesity are likely to lose weight and that longer enrollment duration could potentially lead to greater weight loss. We believe these results to be clinically significant as 5% loss of initial weight has been linked to improved health outcomes among people who are obese, with prediabetes, or type 2 diabetes [9,12,29,30].

These results are in line with previous studies that found digital nutrition interventions to be successful in weight loss [31,32]. The majority of prior studies on digital apps has focused on nutrition monitoring and reporting or health coaching. However, the tools of the Foodsmart platform are unique in that, in addition to dietary recommendations, the app offers a personalized meal and recipe planning program and a unique food purchasing environment that addresses barriers to healthy eating by offering healthy options for everyone such as online ordering and delivery of groceries, meal kits, and prepared foods. Meal planning and cooking at home have been found to be associated with better diet quality and lower likelihood of obesity, primarily due to having control of ingredients, cooking methods, and portion sizes [22,23]. Although the use of commercial online grocery shopping, delivery, and meal kits has been increasing in recent years, few studies have examined the impact of these new purchasing behaviors on health outcomes. A study on medically tailored meal delivery for patients with diabetes and food insecurity found that home delivery of 10 meals per week for 12 weeks was associated with improvements in Healthy Eating Index Score, food insecurity, and hypoglycemia [33]. However, this was a short-term study with direct food provisions, which do not precisely mirror the Foodsmart platform. Nonetheless, the study suggests that healthy food delivery may be a viable strategy in improving health outcomes. More research is warranted to evaluate the potential cost savings of these types of programs that change the food purchasing environment to create healthier eating.

The finding that change in Healthy Diet Score was the strongest predictor of achieving 5% weight loss is noteworthy. The Healthy Diet Score captures overall dietary quality and serves as a proxy for engagement with the Foodsmart platform since the program is designed to improve diet quality. This demonstrates that participants who used the Foodsmart program and improved their diet quality were more likely to lose weight. Furthermore, the association between change in Healthy Diet Score and weight loss was compounded by duration of the program (Figure 3D). This finding is in agreement with previous studies that have found weight loss from mobile health apps to be greater with longer enrollment duration [34]. However, these findings showed that among people who used the program for over 12 months, 5% weight loss was achieved by one-third of users. Previous studies have shown that long-term maintenance of weight loss is challenging as more than half of lost weight was regained within 2 years [35,36]. Although this study was not designed to examine whether weight loss was sustained, we found that longer enrolment duration was associated with greater weight loss. Additional research is needed to further examine the sustainability of this type of intervention by examining trends of multiple weight measurements over time.

We found that despite more females using the program compared to males, that on average, male users lost more weight compared to females when stratified by enrollment length and gender (Figure 3A). However, in the fully adjusted logistic regression model, we did not find a statistically significant association between gender and 5% weight loss. While other studies have also found that males lost more weight compared to women when using health apps [34], the reason for the greater percent change could be higher baseline weight in male users. It was also interesting that when mean percent weight change was stratified by enrollment duration and age category, participants who were older consistently lost more weight, and the effect compounded with longer duration of enrollment. For participants under age 30, on average, weight increased if they enrolled for longer than 12 months. This finding was contrary to what some might expect given high rates of technology use by younger adults [37]. It may be that meal planning and food purchasing interventions may be more successful among older adults, or they may be more focused on their health. Or, this may be due to the increasing trend of eating out and less cooking, leading to weight gain, among younger populations [38,39]. Additionally, younger users may have been more likely to disengage with the app and then re-engage after gaining weight.

There are several limitations of the present study. Due to the observational nature of this study, we cannot conclude any causal associations between change in diet quality and weight loss. Since we did not have a control group, it is difficult to attribute a weight loss to the Foodsmart platform itself. This study serves as an exploration in which factors are associated with weight loss among Foodsmart users. Since we did not have exact dates for leaving the program, we used the last entry of weight as a proximal end date. Because we are using real-world data rather than the settings of a controlled study, participants were free to start and stop usage of the app as they wished. Therefore, it is challenging to draw firm conclusions on how duration of usage was associated with weight loss. Another limitation is that measures of height, weight, and diet were self-reported by participants. However, prior studies suggest that there is moderate to high agreement between online self-reported and measured anthropometric data [40]. Unfortunately, we did not have information on other factors that may be important predictors of weight loss such as total energy intake, race, or socioeconomic status. We did not assess engagement level or usage among participants since our goal was to examine the overall Foodsmart program. All users in this analysis took and retook the Nutriquiz and weight change assessments, which may, in and of themselves, have driven an impact due to their ability to motivate and drive self-insight and knowledge about nutrition and to track progress.

This study also had several strengths. With almost 9000 participants, this study included a large number of participants with obesity that provided us with sufficient power to examine percent weight change stratified by 2 variables. We also had a broad range of enrollment lengths, allowing us to examine weight change and maintenance in a time span of more than 2 years. Few studies, especially randomized clinical trials, on digital apps have follow-up data for weight change after more than 2 years.

In conclusion, this was one of the first studies of this scale and time length to examine weight loss among individuals with obesity who were users of a digital nutrition platform with personalized dietary recommendations and online meal planning, food ordering, grocery discounts, and incentives. Future studies are warranted to determine the sustainability and cost-effectiveness of weight loss through a digital nutrition intervention with these features vs other alternatives. Randomized clinical trials are needed to tease out causal associations.

## Appendix

Multimedia Appendix 1 Major changes to the Foodsmart platform since 2013.

## Abbreviations

AHEI-2010: Alternative Healthy Eating Index-2010
ANOVA: analysis of variance
CSIRO: Commonwealth Scientific and Industrial Research Organization
DPP: Diabetes Prevention Program
