# Supplementary material for: Weight Reduction Through a Digital Nutrition and Food Purchasing Platform Among Users With Obesity: Longitudinal Study
Source: J Med Internet Res. 2020 Sep 2;22(9):e19634. doi: 10.2196/19634 (PMC7495263; doi:10.2196/19634)
Supplement: Multimedia Appendix 1 [file jmir_v22i9e19634_app1.docx]

Multimedia Appendix A. Major changes to the Foodsmart platform since 2013.

| Year | Number of participants enrolled at the time | Major Change to Foodsmart platform |
| --- | --- | --- |
| 2013 | 4 | Addition of Nutriquiz  Addition of Meal Planning feature |
| 2014 | 17 | Major web redesign |
| 2015 | 45 | Addition of social favoriting/commenting of recipes |
| 2016 (end) | 720 | Addition of online food ordering on web |
| 2017 | 1,633 |  |
| 2018 | 4,690 | Major new mobile version (iOS and Android) |
| 2019 | 3,537 |  |
| 2020 (Jan-Apr) | 362 |  |
